# From cold to hot: mechanisms of hyperthermia in modulating tumor immunology for enhanced immunotherapy

**DOI:** 10.3389/fimmu.2025.1487296

**Published:** 2025-02-28

**Authors:** M. Marc Abreu, Alberto F. Chocron, David M. Smadja

**Affiliations:** ^1^ Medicine Department, BTT Medical Institute, Aventura, FL, United States; ^2^ BTT Engineering Department, BTT Medical Institute, Aventura, FL, United States; ^3^ Research Service, Miami Veteran Administration Medical Center, Miami, FL, United States; ^4^ Department of Hematology, AP-HP, Georges Pompidou European Hospital, Paris, France; ^5^ Université Paris Cité, INSERM, Paris Cardiovascular Research Center, Paris, France

**Keywords:** hyperthermia, immunotherapy, immune checkpoint inhibitors (ICIs), heat shock, heat shock protein, CTLA 4, PD1, thermal stress

## Abstract

The emergence of immunotherapies has revolutionized cancer treatment by leveraging the immune system to target malignancies, offering new hope where traditional therapies often fall short. Within this context, hyperthermia (HT) has re-emerged as a promising adjunctive treatment, capable of enhancing the effectiveness of radiotherapy, chemotherapy, and immunotherapy. HT influences both the innate and adaptive immune systems, enhancing the activity of immune cells such as neutrophils, NK cells, and dendritic cells, while also modulating the tumor microenvironment (TME) to promote immunogenic cell death (ICD) and reduce immunosuppressive conditions. These effects contribute to the transformation of immunologically “cold” tumors into “hot” tumors, making them more susceptible to immune-mediated destruction. Furthermore, HT can amplify the efficacy of immune checkpoint inhibitors (ICIs) by improving immune cell infiltration, inducing damage-associated molecular pattern (DAMP) release, and enhancing antigen presentation. Preclinical and clinical studies support the combination of HT with ICIs, demonstrating improved outcomes in otherwise resistant tumors. However, the full therapeutic potential of the different technologies allowing to apply HT remains to be fully understood, and further research is needed to optimize treatment protocols, explore the differential impacts of local versus whole-body hyperthermia, and identify biomarkers for patient stratification. This review underscores the multifaceted role of HT in immunity and its potential to significantly enhance the efficacy of immunotherapy.

## 1 Introduction

Cancer treatment has been revolutionized by immunotherapies, shifting the focus from traditional methods like chemotherapy and radiotherapy to harnessing the immune system to combat cancer (1). These immunotherapies, including immune checkpoint inhibitors (2), adoptive cell therapies (3), and cancer vaccines (4), have shown remarkable efficacy in various cancers, offering new hope where traditional treatments have often failed. The success of immunotherapies highlights the immune system’s vital role in cancer control, targeting cancer’s evasion strategies by enhancing immune cell activity and recognition of tumor antigens. This focus on the interplay between cancer and immunity drives innovative treatments aimed at reactivating immune responses within the tumor microenvironment (TME) (5–7). Within this framework, several tumor classification systems have been developed to more accurately categorize patients based on the distinct properties of their TME (8, 9). Grasping the complexity of TME heterogeneity is vital for crafting effective therapeutic combinations in immunotherapy protocols and for incorporating personalized treatment strategies tailored to individual patient needs.

**Figure 1:**
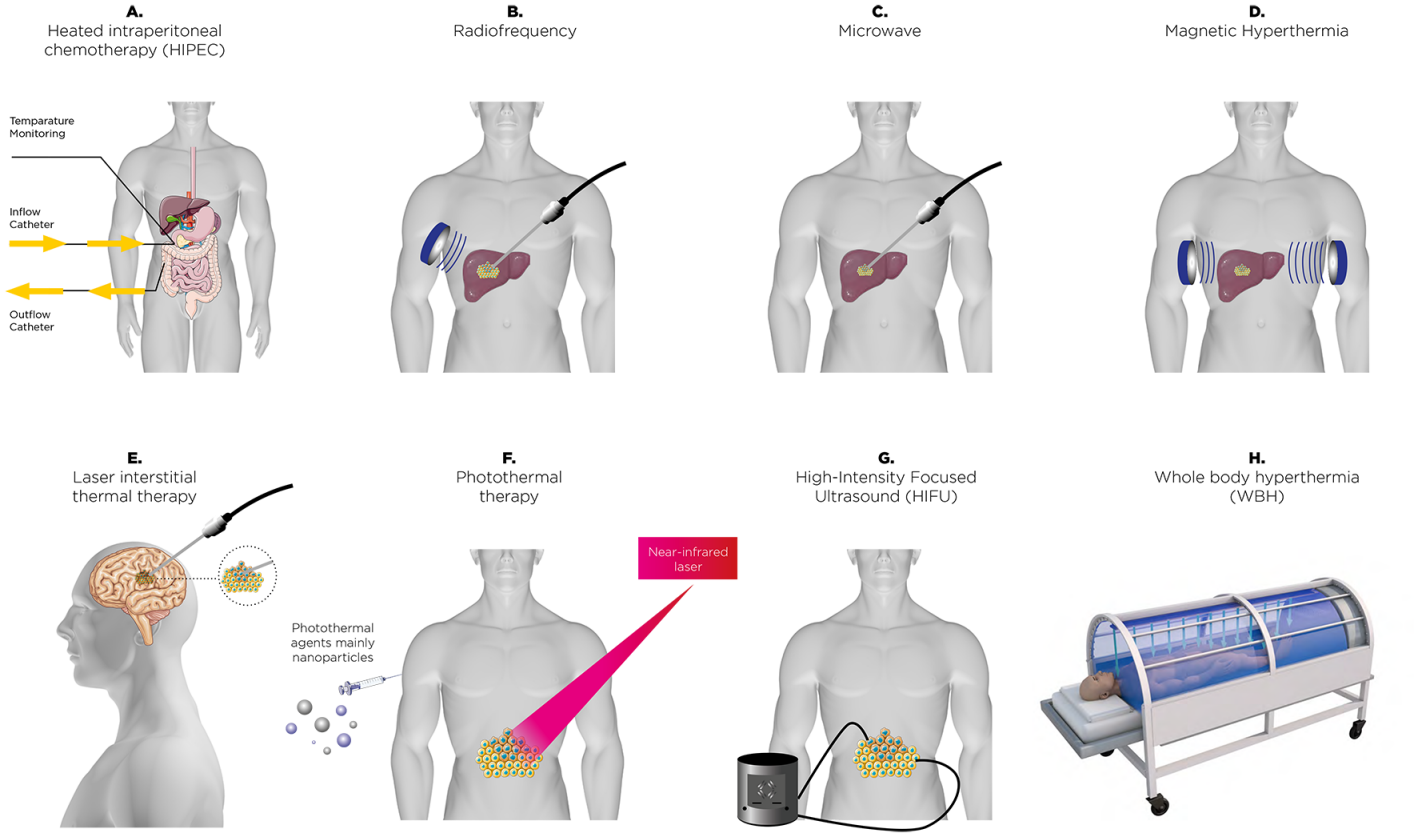
A range of thermal therapies employed in clinical and research contexts to enhance cancer treatment. These approaches include heated intraperitoneal chemotherapy (HIPEC) **(A)**, radiofrequency energy-based ablation **(B)**, microwave-based **(C)**, magnetic field-induced hyperthermia **(D)**, laser interstitial thermal therapy **(E)**, nanoparticle-driven photothermal therapy **(F)**, high-intensity focused ultrasound (HIFU) ablation **(G)**, and systemic whole-body hyperthermia (WBH) **(H)**. Each method uniquely leverages thermal energy for precise or widespread tumor targeting.

Thermal therapy, commonly referred to as hyperthermia (HT), involves deliberately raising tissue temperatures to between 39°C and 42°C for a sustained period, typically around one hour (10–13). The therapeutic benefits of heat have been recognized since ancient times. The earliest documented use of heat treatment can be found in the Edwin Smith Surgical Papyrus, an Egyptian text dating back approximately 5,000 years (14), where a patient with breast cancer was treated using heat. As far back as the fifth century BC, Hippocrates (460–377 BC) observed that malarial fever could alleviate symptoms in epileptics (15, 16). About 150 years ago, physician W. Busch was the first to report the potential benefits of HT in cancer treatment. He observed the regression of a sarcoma following a high fever induced by accidental erysipelas infection (17). A key hypothesis in Thermal Medicine, is that HT induced by externally heating the body or specific tissues without the presence of pyrogenic agents, may provoke a strong thermoregulatory response, which could significantly impact the physiology of the TME and modify the immune response accordingly (18). This approach has been extensively studied, particularly in cancer treatment, where it is frequently combined with radiotherapy, chemotherapy, or immunotherapy to improve treatment efficacy. More recently, HT has emerged as a potential option for managing depression, offering a complementary or alternative method for mood stabilization (19). It is important to differentiate therapeutic HT from other heat-related conditions or treatments. For instance, malignant HT represents a severe, potentially fatal reaction triggered by specific medications (20). Meanwhile, thermal ablation involves heating tissues to temperatures above 44°C with the intent of destroying cancerous cells (21). These distinctions emphasize the unique purpose and temperature parameters of therapeutic HT. Additionally, HT treatments can be further classified based on their method of heat delivery and scope. Heating depth distinguishes the main types: superficial HT targets tissues near the skin’s surface (22), deep HT is designed to treat tissues or organs located further within the body (23), and interstitial HT involves placing heating devices directly into tissues for precise temperature regulation (24). The scale of heating also defines its application: local HT focuses on small, specific areas; loco-regional HT addresses larger zones, such as an entire organ or surrounding tissues; and whole-body hyperthermia (WBH) elevates the body’s overall temperature to induce systemic effects (25). Additionally, HT is categorized based on temperature intensity. Mild HT gently raises tissue temperatures, while moderate HT involves slightly more substantial heating. Fever-range HT mimics the natural temperature increase during fever, potentially stimulating immune activity and providing specific therapeutic benefits. Each of these classifications underscores the versatility of HT as a treatment strategy across various medical technology (**Figure 1**) and applications. HT exerts multiple biological effects that enhance its therapeutic potential, particularly when combined with radiotherapy or chemotherapy. One of the key mechanisms of HT is its ability to induce cell cycle inhibition, primarily by halting cells in the S-phase and G2/M-phase, making them more sensitive to radiation and other treatments (26, 27). HT also disrupts nuclear proteins involved in DNA repair processes, such as BRCA2, which impairs homologous recombination, thereby increasing DNA damage caused by radiotherapy or chemotherapy (28). Furthermore, HT can destabilize cell membranes by altering lipid composition and membrane permeability, leading to increased drug uptake and cytotoxic effects (29). Additionally, HT inactivates proteins responsible for DNA repair, including heat-sensitive enzymes such as PARP1, resulting in an accumulation of DNA damage (30). These effects collectively amplify the efficacy of conventional cancer treatments, highlighting the critical role of HT in enhancing therapeutic outcomes. Thus, alongside these advances in immunotherapy, HT has re-emerged as a promising adjunctive treatment in cancer treatment and may have the potential to amplify the effectiveness of radiotherapy, chemotherapy, and immunotherapy (13). This amplification occurs through various mechanisms, such as boosting blood flow within the TME, which supports lymphocyte infiltration and increases oxygen levels, as well as improving drug delivery (18). The induction of heat shock proteins (HSPs) can also trigger apoptosis or necrosis in cancer cells, leading to alterations in surface marker expression and the release of cellular debris, which act as antigens and stimulate an antitumor immune response (31, 32). These heat-induced cellular stress responses not only promote the direct killing of cancer cells but also enhance the visibility of these cells to the immune system, thereby facilitating the activation of dendritic cells and subsequent T-cell-mediated immunity (31, 32). Moreover, there is growing interest in the potential of HT to modulate the immune system itself (33, 34). These immunomodulatory effects indicate that HT may enhance the efficacy of radiotherapy (RT), chemotherapy (CT), and immunotherapies, as outlined in **Table 1**, which provides a non-exhaustive list of relevant preclinical studies (35–44), potentially enhancing their efficacy or overcoming resistance mechanisms (45, 46). Growing interest in hyperthermia has led to numerous randomized clinical trials (47–52) investigating diverse approaches to its application. These efforts provide a robust foundation for proposing a detailed exploration of its potential mechanisms and therapeutic strategies. In this context of immune transformation after HT, the TME is altered and can shift from “hot,” with high immune infiltration and pro-inflammatory activity, to an even more immunostimulatory state by enhancing T-cell function, trafficking, and heat shock protein expression (53). In contrast, “cold” tumors are characterized by minimal immune cell infiltration and an immunosuppressive environment, which makes them less responsive to immunotherapies. HT can transform these “cold” tumors by increasing immunogenicity, promoting dendritic cell activation, and remodeling the stroma to allow immune cell infiltration, thereby enhancing their susceptibility to immune-based treatments (34, 54).

**Table 1:** A curated selection of preclinical studies showcasing the transformative potential of hyperthermia (HT) in reshaping immune responses and advancing cancer therapy.

Treatment	Mechanistic Insights	Tumor Model
Radiotherapy + HT (35, 36)	increased in tumor cell death induction	MCF-7 and MDA-MB-231
	Increase expression of immune suppressive checkpoint molecules	
Radiotherapy + HT (37)	immune suppressive (PD-L1, PD-L2, HVEM) and immune stimulatory (ICOS-L, CD137-L and Ox40-L) ICMs were significantly increased in glioblastoma cells	U87 and U251
Chemotherapy + HT (38)	conversion of non-inflamed tumors into inflamed ones, with post-treatment immune activity predicting outcomes. Adding hyperthermia enhances the tumor environment, boosting immune responses in high-risk sarcomas.	Immune infiltration in patients’ biopsies
Anti-PD-1 and HT (Radio-frequency ablation) (39)	Reduced tumor size; Prolonged survival rates; Strong systemic immune response.	CT26 and B16
Anti-PD-1 and HT (Photothermal therapy) (40)	increase in lymphocytes infiltration and inflammatory cytokine, prevent distant metastasis and survival	CT26 and 4 T1 cells
Anti-PD-1 + Anti-CTLA-4 + HT (Magnetic hyperthermia) and Radiotherapy (41)	Reduced tumor size and metastasis; Improved systemic anti-tumor response.	4T1-luc breast tumor
Anti-PD-L1 and HT (Magnetic hyperthermia) (42)	Reduced tumor volume; Enhanced immune system memory; Effective control of metastatic progression.	4T1 breast tumor
Anti-PD-L1 and HT (Photothermal therapy) (43)	prevent and inhibit metastasis by a long-term immune memory and increased survival	4T1 and B16F10 cells
anti-PD-1 and anti-CTLA-4 antibody treatment (ipilimumab/nivolumab) with IL-2 under taurolidine protection and locoregional hyperthermia and whole body hyperthermia (WBH) (44)	Complete Clinical Remission of a stage IV triple negative breast cancer with lung metastasis	case report

This non-exhaustive list highlights the pivotal role of HT in immunomodulation and its synergistic effects with cutting-edge treatment strategies.

This review explores the intricate interactions between HT and the immune system, a promising and emerging field with the potential to reshape cancer therapy. By providing a comprehensive overview of current knowledge, we aim to highlight how HT influences immune responses and its implications for advancing cancer treatment strategies.

## 2 Heat shock protein, hyperthermia and immunotherapy

**Figure 2:**
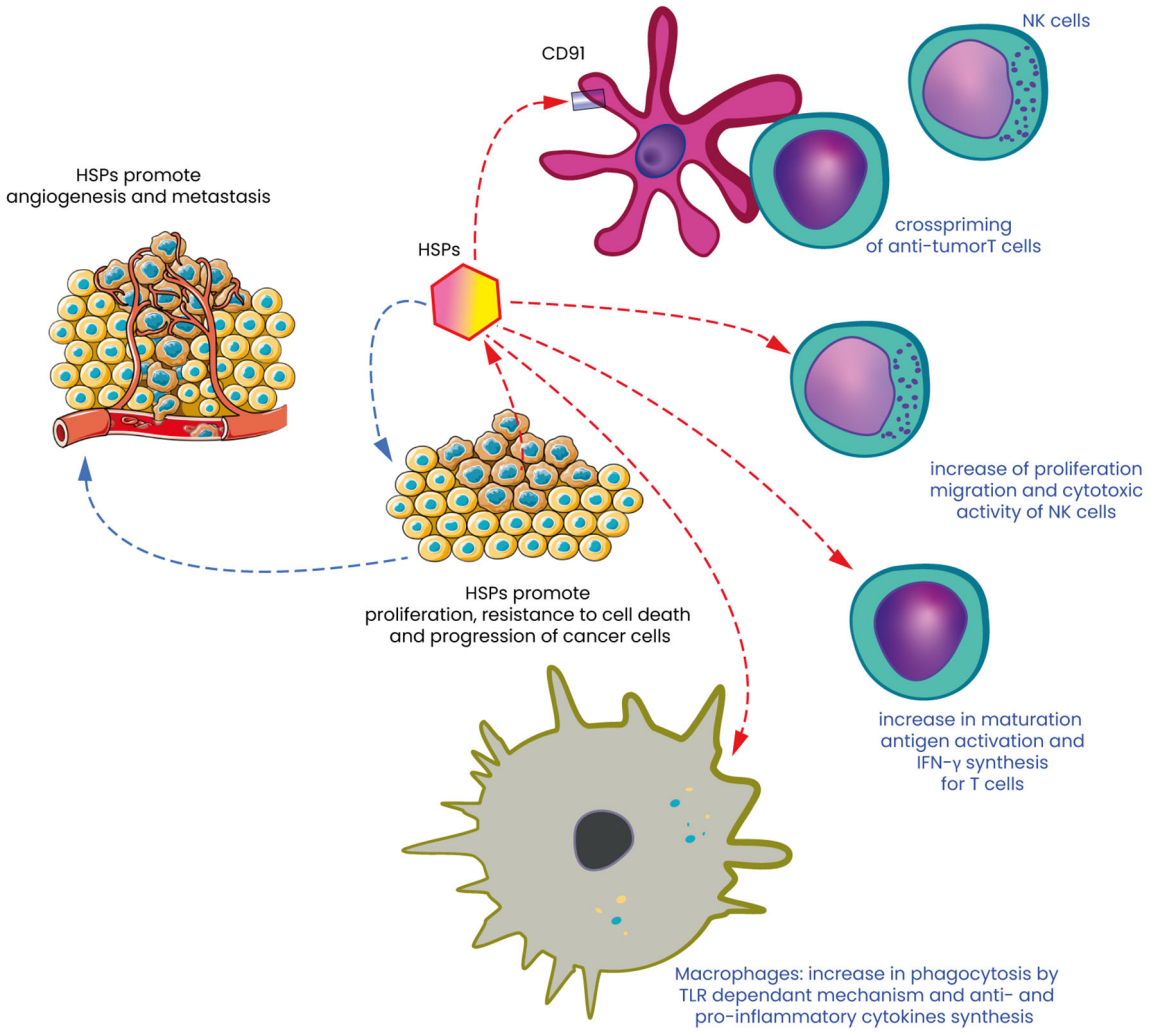
Heat shock proteins: the double-edged sword of cancer cell survival and immune response. Heat Shock Proteins (HSPs) are molecular chaperones that play a crucial role in maintaining cellular homeostasis by assisting in the proper folding of proteins and preventing aggregation under stress conditions. In the context of cancer, HSPs act as a double-edged sword. On one hand, they contribute to cancer cell survival, promoting tumor progression and angiogenesis (left part of **Figure 1**) and on the other hand, HSPs can also potentiate the immune response against tumors and destroy these same cells. They serve as danger signals that are recognized by the immune system, leading to the activation of dendritic cells (DC) in particular by activating CD91 and the subsequent presentation of tumor antigens to T cells. HSPs can also act as immunogenic molecules, enhancing the recognition and destruction of cancer cells by NK cells. HSPs are often expressed on the surface of stressed or damaged cancer cells, where they can bind to receptors on NK cells, such as NKG2D. This binding triggers the activation of NK cells, leading to the release of cytotoxic granules and the destruction of the target cancer cells. Moreover, extracellular HSPs can act as danger signals, promoting the recruitment and activation of NK cells within the TME, thereby contributing to the anti-tumor immune response. Finally, macrophages, another crucial component of the innate immune system, are influenced by HSPs by interacting with macrophages by binding to Toll-like receptors (TLRs) and other pattern recognition receptors on the surface of these immune cells. This interaction can lead to phagocytosis but also to the activation of macrophages in a pro-inflammatory (M1) phenotype, which supports anti-tumor immunity.

HT, along with various other stress conditions, triggers the production of heat shock proteins (HSPs), which are essential for cellular protection and survival (55). These proteins are grouped into different families based on their molecular weight, and they function as molecular chaperones. The heat shock response (HSR) is structured as a sequential process, where information is transmitted through the localized activity of molecular chaperones (8). This means they assist in stabilizing and repairing damaged proteins, preventing harmful interactions between misfolded proteins, and aiding in the removal of defective proteins from the cell. The primary families of heat shock proteins (HSPs) encompass both small and large molecular weight groups. These include small HSPs like HSP27, as well as larger HSPs such as HSP47 (56, 57), HSP70 and HSP90 (58). Additionally, the human chaperonin families, including HSP60/HSP10, are also part of these main categories. Among the various HSPs, HSP27 and HSP70 are particularly notable for their ability to protect cells from potentially lethal stimuli by enhancing resistance to apoptosis and promoting cellular homeostasis. The synthesis of HSPs is generally upregulated in response to HT, a process that enables cells to cope with elevated temperatures. However, at extremely high temperatures, the production of HSPs is inhibited, which can lead to cell death. Under normal physiological conditions, HSPs play a critical role in maintaining cellular integrity, particularly by protecting cells from damage induced by stress and by enhancing their survival capabilities (32). While the intracellular functions of HSPs are well-documented, their roles in the extracellular environment are equally important, particularly in the context of cancer and the immune response (32, 59, 60). In cancer, HSPs are often overexpressed, which contributes to tumor development, progression, resistance to therapy and angiogenesis (61–64). This overexpression is associated with several processes, including the inhibition of apoptosis, the promotion of cell proliferation, and the enhancement of metastatic potential. For example, HSP70 and HSP90 are frequently upregulated in tumors, where they help stabilize oncogenic proteins, thereby supporting cancer cell survival and growth (63, 64). Extracellular HSPs have garnered significant attention for their involvement in the immune response, particularly in the context of cancer (65, 66). When released into the extracellular space, these proteins can function as danger signals, signaling the immune system to recognize the existence of damaged or stressed cells., including cancer cells (**Figure 2**). This alert system is crucial for initiating and coordinating an immune response against these aberrant cells. One of the primary mechanisms through which extracellular HSPs influence the immune system is by facilitating the cross-presentation of tumor antigens. HSPs such as gp96, HSP70 and HSP90 can chaperone tumor-derived peptides and deliver them to antigen-presenting cells (APCs), including dendritic cells and macrophages (**Figure 2**). This process is essential for the activation of cytotoxic T lymphocytes (CTLs/CD8^+^ T cells), which are the immune cells responsible for targeting and destroying cancer cells. The interaction between HSPs and APCs is often mediated by specific receptors, such as CD91, which plays a crucial role in the uptake of HSP-peptide complexes and the subsequent presentation of these peptides on MHC class I molecules (67–71). This pathway of antigen presentation is particularly important in the context of tumors, where the immune system’s ability to recognize and respond to cancer cells is often impaired (32, 65). By enhancing the visibility of tumor antigens to the immune system, extracellular HSPs can help restore immune surveillance and promote the destruction of cancer cells. This process is vital in cancer therapy, where the goal is to elicit a robust immune response against the tumor. The dual role of HSPs in cancer—acting both as protectors of tumor cells and as modulators of immune responses—presents a unique challenge but also offers promising opportunities for therapeutic intervention.

## 3 Hyperthermia and innate immunity

HT significantly enhances the activity of innate immune cells, such as neutrophils, natural killer (NK) cells, monocytes/macrophages, and dendritic cells.

Fever-range HT have been associated to activation and bactericidal function of neutrophils (72, 73). Thermal stress also promotes increased neutrophil recruitment to tumors (72). This thermal stress-induced neutrophil migration is partly driven by heat-induced elevations in circulating neutrophils, which are dependent on granulocyte colony-stimulating factor (G-CSF). G-CSF plays a pivotal role in a model of radiation-induced neutropenia, where fever-range WBH significantly accelerates neutrophil recovery in the bloodstream and increases the number of hematopoietic stem cells and neutrophil progenitors in the bone marrow (74). However, the effects of HT are highly dependent on the specific heating protocols and the localization of recruited cells (75).

Recent findings confirm that hyperthermia enhances the cytotoxic activity of natural killer (NK) cells and their mobilization, improving their ability to recognize and eliminate tumor cells, addressing previous uncertainties surrounding this effect (76). Indeed, HT at varying temperatures might indicate that NK cell activity and cytotoxic T-cell function are enhanced at moderate temperatures like 40°C (77). Studies have shown that therapeutic WBH leads to reversible changes in lymphocyte subpopulations, such as an increase in NK cells, NKT cells, and γδ-T cells, all of which are associated with innate immune functions (78).

Moreover, the myeloid-derived suppressor cells (MDSCs) are often recruited to the TME, where they support tumor progression by suppressing anti-tumor immune responses (79, 80). MDSCs are often recruited to the TME, where they support tumor progression by suppressing anti-tumor immune responses. Given their central role in promoting immune suppression in cancer and other diseases, MDSCs have become an important target for therapeutic intervention. HT, applied through various *in vitro* or *in vivo* methodologies, has been demonstrated to affect MDSCs in multiple ways, including decreasing their recruitment to the TME (81–86).

HT induces the release of damage-associated molecular patterns (DAMPs), such as extracellular HSP70, which not only diminish the immunosuppressive activity of MDSCs but also enhance CD4+ T cell-mediated anti-tumor responses. This effect has been highlighted in studies by Zhu et al. (84, 87). Additionally, the use of low-dose β-adrenergic receptor blocker therapy, such as propranolol, has been identified as a complementary approach to reducing MDSC accumulation (85). Propranolol mitigates physiological stress responses by blocking β-adrenergic signaling, thereby decreasing stress-induced recruitment of MDSCs. This finding underscores the potential for integrating stress-reducing interventions with thermal treatments to optimize anti-tumor immunity. Moreover, HT drives the secretion of pro-inflammatory cytokines such as CXCL10 and IL-6, which create an immune-supportive environment that counters MDSC-mediated suppression (83). Finally, Extracellular vesicles (EVs) released during hyperthermia (HT) have been demonstrated to significantly impact the immune landscape by decreasing MDSC recruitment and promoting anti-tumor immunity. The study by Cen et al. (88) delves into the role of serum-derived extracellular vesicles (sEVs) released following cryo-thermal therapy in reducing MDSC-mediated immunosuppression and enhancing therapeutic outcomes (88).

## 4 Inflammatory cytokines, biomarkers and Hyperthermia

HT can influence the expression and function of proinflammatory cytokines (89), which play a critical role in regulating antitumor immune responses. WBH has been demonstrated to stimulate the release of various pro-inflammatory cytokines (78, 90, 91). HT has been shown to enhance IL-1-induced T-cell proliferation, demonstrating that IL-1’s function is highly responsive to thermal changes. In addition to its sensitivity to increased temperature, IL-1 also plays a crucial role in raising body temperature during fever (92). Another study demonstrated that a slight increase in body temperature to 39.5°C in mice exposed to total body irradiation led to improved recovery from neutropenia (74, 93). This recovery was propelled by a heat-induced cytokine cascade that significantly increase neutrophil production (93). Moreover, tumor samples from a group of 22 pet dogs with naturally occurring soft tissue sarcomas who underwent thermoradiotherapy demonstrated that before and 24 hours after the initial HT session alterations in the water diffusion coefficient, a marker for inflammation, were associated with changes in various inflammation-related genes (94). Additionally, systemic HT has been shown to influence the activity of the proinflammatory cytokine IL-6 within the TME, potentially diminishing its role in tumor progression (95). Indeed, HT has been propose to counteract the protumorigenic effects of IL-6 by promoting the trafficking of effector T lymphocytes to the TME (96). They found that neutralizing IL-6 prevented the selectin and Intercellular adhesion molecule-1 (ICAM-1) – dependent migration of adoptively transferred CD8^+^ T cells through the tumor vasculature (96). Furthermore, in IL-6-deficient mice, HT increased ICAM-1 expression on tumor vessels and induce CD8^+^ T-cell infiltration into the tumor (96). Temperatures around 42°C, appear to induce a temporary shift in lymphocyte function towards an anti-inflammatory state. This is evidenced by a significant rise in plasma IL-10 levels, coupled with a decrease in IL-12 and IFN-γ during and shortly after treatment (97). Moreover a marked increase in serum levels of sIL2-R has been observed, indicating significant T-cell activation (78). More recently, studies suggest that fever-range WBH could have significant impacts on the immune system, enhancing responses such as antigen-specific T-cell responses. Indeed, in 5 healthy volunteers, exposure to fever-range HT (38.5°Celsius for 60 minutes) has been shown to stimulate immune responses, specifically in T-cells. Kobayashi et al. demonstrated that exposure to physiologically relevant thermal stress can significantly boost cytokine production in human peripheral T cells, enhancing their sensitivity and response to specific antigens (98). In the study, volunteers underwent WBH, during which their rectal temperature was elevated and maintained above 38.5°C for over an hour. Peripheral blood mononuclear cells (PBMCs) were sampled both before and after this thermal treatment. The induced thermal stress appeared to increase membrane fluidity in T cells, potentially accelerating and optimizing the clustering of molecules essential for antigen recognition and signal transduction, thereby amplifying T-cell activation and immune efficiency (98).

Sulyok et al. conducted a randomized trial evaluating the impact of preoperative fever-range (FR) whole-body hyperthermia (WBH) on immune markers in colorectal cancer surgery patients (99). Their findings revealed that the FR-WBH group exhibited a significant increase in heat shock proteins (HSPs), particularly HSP60 and HSP90, compared to the control group, while HSP70 levels remained unchanged. Interestingly, tumor necrosis factor-alpha (TNF-α) levels surged post-surgery in the control group but remained near baseline in the FR-WBH group, suggesting a protective immune-modulatory effect of hyperthermia (99). These findings align with earlier studies emphasizing the immunostimulatory properties of fever-range hyperthermia. Additionally, Yu et al. reported that HT-induced immune activation might influence long-term survival in rectal cancer patients, reinforcing the potential of HT as an adjunctive immunotherapeutic strategy (100).

## 5 Hyperthermia, heterogeneous regulation of lymphocytes subpopulations and adaptative immunity

**Figure 3:**
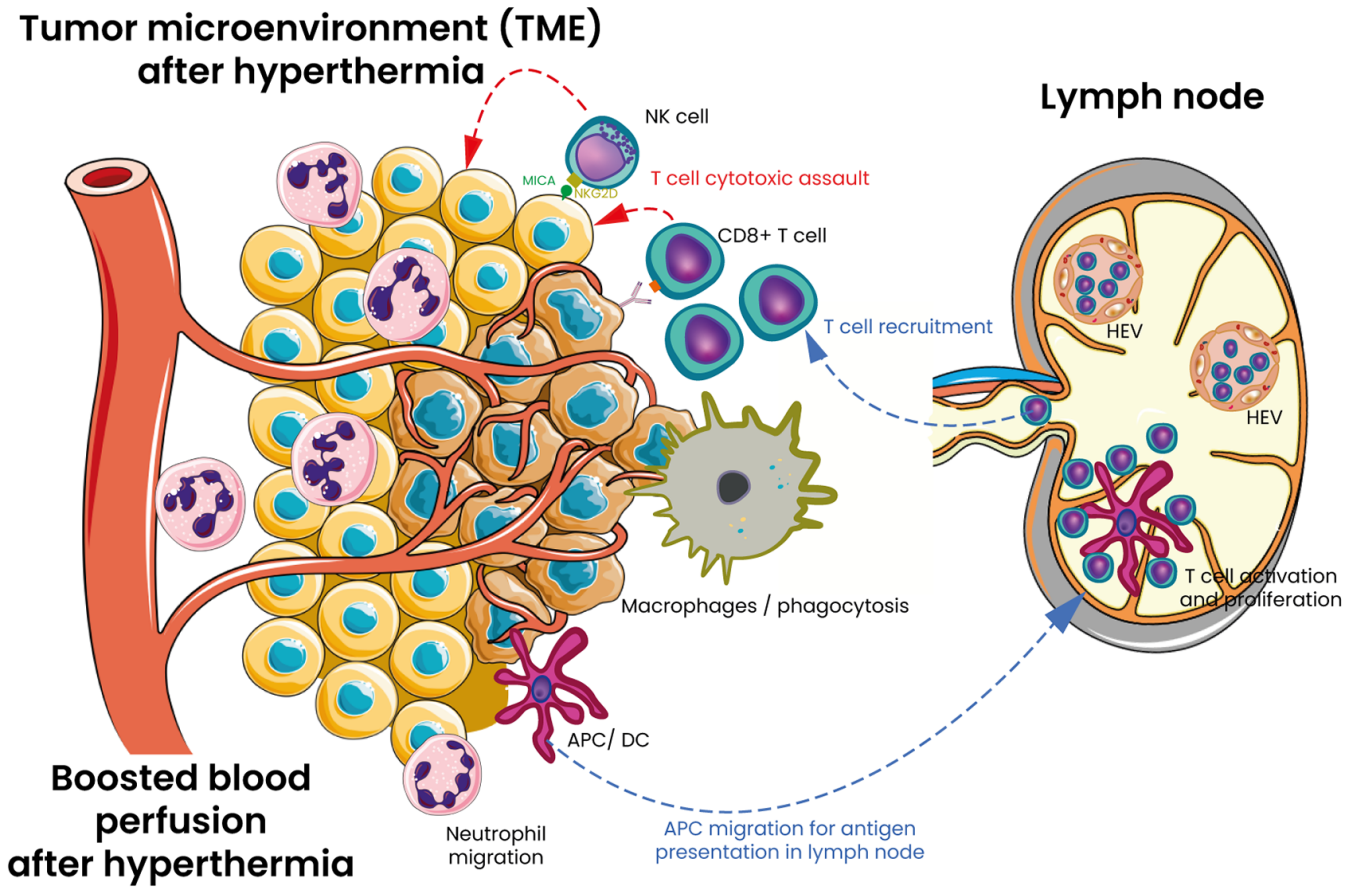
Mechanisms of HT on innate and adaptative immunity in tumor TME. HT serves as a versatile adjuvant that modifies the TME through several pathways. It enhances vascular perfusion and blood circulation within the tumor. Additionally, HT promotes the trafficking of CD8^+^ T cells by inducing the expression of E/P selectin and intercellular adhesion molecule 1 (ICAM-1) on tumor-associated blood vessels. It also boosts T-cell receptor (TCR) signaling and facilitates the differentiation of naïve T cells into effector cells and at the end cytotoxic attack of tumor cells. Furthermore, HT enhances the expression of MHC class I ligand (MICA/B) on tumor cells and upregulates the NKG2D ligand on NK cells, thereby increasing the cytotoxic potential of NK cells. The functional activity of macrophages and dendritic cells is also elevated which stimulates further immune responses and enhances antigen presentation. HT enhances neutrophil degranulation, phagocytic activity, and antigen presentation by M1 macrophages and dendritic cells. Following antigen uptake, dendritic cells travel to lymph nodes, where they display tumor antigens to T cells, initiating the proliferation of cytotoxic T cells. These cytotoxic T cells (CD8^+^ T cells), along with natural killer (NK) cells, then infiltrate tumors and target cancer cells for destruction by releasing cytotoxic granules and activating the Fas-FasL pathway. HT also enhances adaptive immune responses by accelerating lymphocyte movement through high endothelial venules (HEVs) in peripheral lymph nodes by modulating different stages of the adhesion process. Moreover, HT directly affects HEVs, promoting the shift from transient rolling to firm arrest of lymphocytes by increasing the density of several adhesion molecules on the vascular surface.

HT has been described to activate lymphocytes. Indeed, initial studies suggested that applying WBH at 41.8-42.2°C in patients with advance cancer, led to a reduction in CD4^+^ T-cells, accompanied by an increase in NK cells and γδ-T cells, which resulted in a decrease in the CD4^+^/CD8^+^ ratio (97). Additionally, a decrease in T-lymphocyte counts was also noted in cancer patients receiving therapeutic WBH at temperatures ranging from 39° to 40°C (101). Moreover, lymphocyte apoptosis could also play a crucial role in immune regulation, and HT appears to influence this process through multiple pathways beyond direct thermal damage. First, HT could induce a stress-related apoptosis of lymphocytes (102, 103) at least in part through a Fas dependent mechanism (104). During WBH, increased lymphocyte apoptosis has been reported, mainly involving CD4^+^ T cell (105). This is likely due to direct heat damage, suggesting that, at least in part, this phenomenon is stress-related. Second, HT can induce immunologically mediated apoptosis (106). Apoptosis is not only a response to stress but also a crucial immune regulatory mechanism. During immune development, T and B lymphocytes undergo programmed cell death if they fail to meet certain functional criteria, such as lacking a functional antigen receptor at various stages of their development, maintenance, and activation (107, 108). Later in their maturation, lymphocytes that exhibit overly strong or insufficiently weak interactions with antigens are also eliminated through apoptosis, a process that helps prevent autoimmune responses and its regulation in the immune microenvironment (109). This form of apoptosis is regulated by several factors, including nuclear steroid hormone receptors like NUR77, which influence both transcriptional programs and mitochondrial function by releasing cytochrome c (107). Heat has been demonstrated as a influencer of lymphocyte trafficking since a transient reductions in circulating T cells has been observed in mice or cancer patients FR-WBH (75, 96, 101). Subsequent research demonstrated that applying direct heat to T or B cells enhanced their adhesion to high endothelial venules (HEVs) and improved their homing capabilities to lymph nodes. This increased homing of lymphocytes from the bloodstream to tissues has been associated to upregulated expression of L-selectin and α4β7 integrin (96). The heat-induced upregulation of these molecules suggest that FR-HT can effectively mimic inflammatory conditions to support stable lymphocyte adhesion and migration (110). Once inside lymphoid organs, lymphocytes exposed to febrile temperatures exhibit an enhanced capacity to respond to stimulatory signals. Exposing T cells directly to heat significantly boosts their proliferation when stimulated by mitogens (111). Additionally, heat activated CD8^+^ T cells demonstrate greater differentiation towards an effector phenotype, including reduced L-selectin expression, increased cytotoxic activity, and higher IFNγ production (112, 113). Heat-induced alterations in membrane fluidity and molecular organization are also seen in CD4^+^ T cells, effectively reducing the dependency on CD28 co-stimulation for IL-2 production (114). These results suggest that HT could potentiate T cell activation by lowering the activation threshold and speeding up effector T cell differentiation. The question now is whether the enhanced immune response, along with broader effects on the TME, plays a significant role during HT treatment. Mechanisms of HT on innate and adaptative immunity are shown and outlined in **Figure 3**.

## 6 Using heat to modify tumor microenvironment

**Figure 4:**
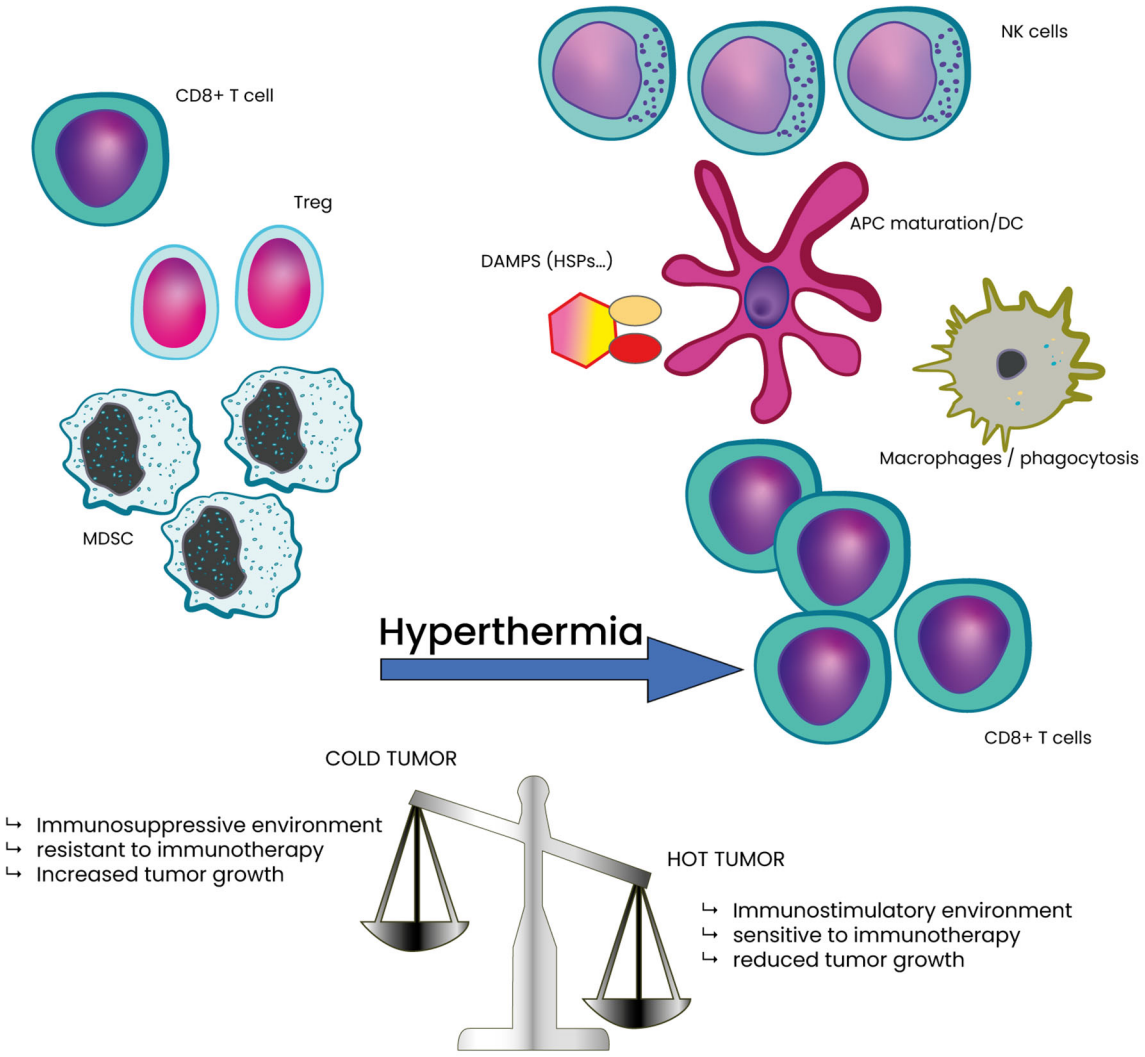
Classification of tumor as cold or hot tumor based on the TME composition in immune cells and potential effect of HT. HT stress shifts the TME towards a less immunosuppressive state. This shift is marked by a significant decrease in intra-tumoral myeloid-derived suppressor cells (MDSCs) and regulatory T (Treg) cells, along with a corresponding increase in CD8^+^ T cells, compared Hot tumor also contributes to lower tumor growth and decreased tumor cell survival.

The TME is a complex ecosystem comprising immune cells, stromal cells, blood vessels, extracellular matrix, and signaling molecules, all of which influence tumor progression and therapy response (5, 115). A key feature of the TME is hypoxia, resulting from rapid tumor growth that outpaces oxygen supply (116). Hypoxia fosters an immunosuppressive environment by impairing cytotoxic T-cell activity and recruiting suppressive cells like regulatory T cells and myeloid-derived suppressor cells (34, 116–118). It also promotes angiogenesis via VEGF-A upregulation and drives tumor survival through hypoxia-inducible factors (119–121). The TME is also crucial in defining tumors as “hot” or “cold” (**Figure 4**) (122–125). Hot tumors are highly infiltrated by CD8+ T cells and express checkpoint molecules (e.g., PD-L1), making them more responsive to immunotherapy (9). Cold tumors, in contrast, have low immune infiltration and are dominated by suppressive cells such as regulatory T cells and MDSCs, leading to immune evasion and reduced immunotherapy efficacy (9). Cold stress can further modulate immune activity in the TME (126). It drives macrophages toward alternative activation via IL-4 and IL-13, leading to norepinephrine production (127). Cold exposure also reduces dendritic cell-mediated T-cell activation and promotes an immunosuppressive TME characterized by increased MDSCs and regulatory T cells, while decreasing CD8+ effector T cells, ultimately accelerating tumor progression (126, 128–130).

HT has the potential to significantly influence both hypoxia in the TME and the cold/hot tumor classification. It enhances oxygenation through improved blood flow and vascular perfusion while simultaneously modulating immune responses by increasing immune cell infiltration and activity. These effects can convert cold tumors into hot, immunologically active tumors, making them more responsive to therapies.

### 6.1 HT and tumor oxygenation

HT modifies the tumor vasculature by improving blood flow and oxygenation, which helps reduce hypoxia. When tumors are exposed to heat, oxygen levels increase and this reoxygenation is associated with enhanced tumor sensitivity to radiation (131, 132). In canine studies, combining HT with radiotherapy led to a prolonged enhancement in oxygenation of hypoxic tumors, which in turn improved their response to radiation (133). The ability of mild HT to reoxygenate tumors is not only critical for enhancing the effectiveness of radiation therapy but also plays a key role in tumor immunology. Sen and colleagues proposed that heating large areas of normal tissue surrounding a tumor triggers thermoregulatory responses controlled by the nervous system, which increase blood flow to dissipate excess heat (134).

HT also targets tumor cell metabolism, influencing hypoxia. For instance, heat-induced activation of hypoxia-inducible factor 1 (HIF-1), a key transcription factor in oxygen homeostasis, along with its downstream targets, such as vascular endothelial growth factor A (VEGF-A) and 3-phosphoinositide-dependent protein kinase 1 (PDK1), leads to improved tumor vascularization and oxygenation (135, 136). Finally, improved oxygenation of the tumor following HT may enhance the eradication of cancer stem cells and increase their sensitivity to both radiotherapy and chemotherapy (137).

### 6.2 HT and immune system activation

Apart from improving oxygenation, HT enhances immune responses by increasing the infiltration and activation of immune cells, such as T and NK cells. Strategies to convert “cold” tumors into “hot” ones are an area of active research to improve the effectiveness of immunotherapies in these types of tumors. HT has the potential to transform non-immunogenic “cold” tumors into immunogenic “hot” tumors by inducing immunogenic cell death (ICD), thereby enhancing the antitumor immune response (138). By increasing the temperature within the TME, HT can initiate the release of danger-associated molecular patterns (DAMPs) from dying tumor cells, which subsequently attract and activate immune cells (138). This thermal intervention not only enhances immune cell recruitment but also re-polarizes immunosuppressive M2 macrophages into pro-inflammatory, anti-tumor M1 macrophages (139). This re-polarization process plays a crucial role in inducing ICD, which in turn activates dendritic cells (DC), T lymphocytes, and natural killer (NK) cells, leading to a robust antitumor immune response and contributing to the inhibition of tumor growth. Consequently, HT can convert previously immune-silent tumors into immunogenic ones, making them more recognizable and susceptible to immune system attacks.

### 6.3 HT, nanomedicine, and immunotherapy enhancement

HT, in combination with nanomedicines that generate localized heat and reactive oxygen species (ROS), presents a promising strategy for improving cancer immunotherapy efficacy (138, 140, 141). These nanomedicines can precisely target tumor sites, creating optimal conditions for ICD and enhancing immune-mediated tumor destruction. Consequently, HT and nanomedicine-based approaches hold great potential for transforming cold tumors into hot tumors, making them more susceptible to immune system attacks.

## 7 Harnessing the power of synergy: hyperthermia and radiotherapy unite to amplify anti-tumor immunity

Radiotherapy (RT) remains a cornerstone in cancer treatment, effectively delivering ionizing radiation to destroy tumor cells and shrink tumors. For a long time, radiotherapy (RT) was considered immunosuppressive due to the radiosensitivity of immune cells (142). However, recent evidence shows that RT can activate the immune system through ICD leading to the release of tumor antigens and DAMPs, stimulating immune responses and enabling bystander and abscopal effects (143). ICD promotes the release of key DAMPs like calreticulin (CRT), which facilitates phagocytosis by dendritic cells (DCs), ATP, which recruits and primes immune cells, and HMGB1, which interacts with TLR4 to activate antigen presentation (144). These signals polarize DCs and macrophages, enhancing the uptake and presentation of tumor-associated antigens (TAAs) to CD8+ T-cells in lymph nodes (145). RT also activates the cGAS-STING pathway, where irradiation-induced DNA damage causes cytosolic DNA accumulation (146–149). This triggers cGAS to produce cGAMP, which activates STING and drives type-I interferon (IFN-α/β) production (150). Type-I IFNs recruit DCs, enhance T-cell priming, and improve immune trafficking to the TME (151). Additionally, IFN-γ from activated T-cells upregulates MHC-I on tumor cells, enhancing immune recognition (152). RT further remodels the TME by increasing inflammatory chemokines like CXCL9, CXCL10, and adhesion molecules such as ICAM-1 and VCAM-1, which promote T-cell infiltration and leukocyte migration into tumors (153). However, excessively high doses can activate DNA-degrading enzymes like TREX1, limiting cGAS-STING activation and systemic immune responses (143, 151). Various RT subtypes have been developed to improve precision, dose delivery, and immunological effects. Among these, Stereotactic Body Radiotherapy (SBRT) and lattice radiotherapy are particularly notable due to their ability to enhance immune responses and modulate the TME. SBRT delivers high-dose radiation in 1–5 fractions with sub-millimeter precision to extracranial tumors, such as those found in the lung, liver, pancreas, and spine, while sparing adjacent normal tissues (154). This high-dose, hypofractionated approach induces ICD, releasing tumor-specific neoantigens and DAMPs, such as HMGB1 and ATP. These molecules enhance dendritic cell maturation and antigen presentation to T-cells, ultimately activating the adaptive immune system (155). Through this mechanism, SBRT has demonstrated its potential to trigger abscopal effects, where local irradiation leads to systemic tumor regression at distant, untreated sites (156). When combined with immune checkpoint inhibitors, such as anti-PD-1 or anti-CTLA-4 antibodies, SBRT creates a favorable TME that enhances the efficacy of immunotherapy (157). Lattice radiotherapy is a novel approach that delivers non-uniform radiation doses, creating high-dose “hotspots” (vertices) interspersed with lower-dose regions within large, bulky tumors. The high-dose vertices induce localized ICD releasing neoantigens and pro-inflammatory signals such as HSPs and cytokines (158). Meanwhile, the low-dose regions preserve tumor perfusion, reducing hypoxia and enabling better infiltration of immune cells into the TME (159). Lattice RT also triggers a bystander effect, where immune responses initiated in the high-dose regions spread to the lower-dose areas, amplifying the overall immune response and tumor control (160). This approach can also prime systemic immune activation by increasing the release of neoantigens and inflammatory cytokines, effects that are further enhanced when combined with HT or immunotherapy (161). The combined use of HT and RT has shown clinical benefits across various cancers, including head and neck, melanoma, breast, cervical, and rectal cancers (47–49, 151, 162). Both treatments exhibit complementary and synergistic effects on the immune system, enhancing anti-tumor responses through shared pathways. Together, they stimulate the release of DAMPs, which activate CD8+ T-cells and promote leukocyte trafficking by upregulating cell adhesion molecules. RT and HT upregulate together leukocyte adhesion and improved lymphocyte infiltration into tumors. HT complements RT by causing sublethal damage, enhancing blood flow, and creating a favorable TME that supports immune cell recruitment even at distant metastatic sites (151). Both modalities also upregulate extracellular HSP70, a critical DAMP that boosts macrophage and dendritic cell (DC) recognition and enhances tumor antigen presentation in lymph nodes, leading to more robust cytotoxic T-cell activation. Additionally, the increased tumor cell death observed with combined HT and RT amplifies DAMP release, further stimulating immune responses. The combination of HT and RT holds significant potential to generate stronger systemic anti-tumor immunity compared to either treatment alone, owing to their complementary mechanisms and synergistic effects on immune activation, cell death, and lymphocyte trafficking.

## 8 Combining hyperthermia with immunotherapy: is it a good idea?

### 8.1 Rationale for combining HT and ICIs

Hyperthermia (HT) has emerged as a valuable adjunct in cancer therapy, with increasing evidence supporting its role in enhancing anti-tumor immune responses. When combined with immune checkpoint inhibitors (ICIs) such as PD-1/PD-L1 and CTLA-4 blockers (161, 162), HT has the potential to overcome resistance in tumors that are poorly infiltrated by immune cells, also known as “cold” tumors, as described in the previous chapter of this paper (9). The synergy between HT and ICIs is driven by multiple mechanisms (163, 164), including modulation of the TME, enhancement of immune cell infiltration, and induction of immunogenic cell death (ICD) (45, 46, 165).

### 8.2 HT and ICIs: TME modulation, immune cell infiltration and immunogenic cell death

One of the key mechanisms by which HT enhances the efficacy of ICIs is through its ability to alter the TME. HT improves blood flow and oxygenation, reducing tumor hypoxia, a condition that often supports tumor growth and immune evasion (166). This reoxygenation diminishes immunosuppressive signals within the TME, making tumors more susceptible to immune attack. Additionally, HT promotes the release of danger-associated molecular patterns (DAMPs) from dying tumor cells (**Figure 4**). These signals attract dendritic cells, which are crucial for antigen presentation and help prime the immune system for a stronger response to ICIs.

HT facilitates the infiltration of immune cells into tumors, a critical step in effective immune-mediated tumor destruction (**Figure 4**). Studies have demonstrated that HT increases vascular permeability, allowing CD8+ T cells and NK cells to enter tumors more efficiently. This effect is further supported by HT-induced upregulation of adhesion molecules on endothelial cells (167, 168), which enhances immune cell recruitment and retention at the tumor site. By promoting sustained immune activity, HT improves the long-term effectiveness of ICIs in tumor control.

A crucial aspect of HT’s synergy with ICIs is its ability to induce ICD. Unlike apoptotic cell death, which is typically non-immunogenic, ICD triggers the release of immune-stimulatory molecules that alert the immune system to the presence of dying tumor cells. HT-induced protein denaturation exposes tumor antigens, making them more recognizable to immune cells. This not only enhances HT’s direct cytotoxic effects but also amplifies the efficacy of ICIs by providing a continuous source of tumor antigens for immune recognition and attack (136, 137).

### 8.3 Preclinical evidence supporting HT-ICI combination

Preclinical studies provide strong evidence supporting the combination of HT and ICIs in cancer treatment (45, 46). In murine models of colorectal cancer, HT has been shown to increase the infiltration of CD8+ T cells into tumors, thereby enhancing the efficacy of anti-PD-1 therapy (169). Similarly, in models of pancreatic cancer, a notoriously “cold” tumor (170–172), HT has been demonstrated to improve the effectiveness of ICIs by altering the dense stromal environment that typically hinders immune cell infiltration. Combining HT with anti-PD-1 or anti-CTLA-4 therapy in breast cancer models has resulted in significant tumor regression and improved survival compared to either treatment alone (41, 173–176). In glioblastoma (GBM), also considered an immune-cold tumor like pancreatic cancer (37, 177–179), HT combined with ICIs may shift the immunosuppressive environment into a more immune-responsive state. Gold nanoparticles amplify photothermal ablation, and when paired with anti-PD-L1 therapy, they reduce tumor size, improve survival, and induce long-term immunity (180). Overcoming the blood-brain barrier (BBB) remains a crucial challenge, but magnetic HT therapy (MHT) shows promise in enhancing antibody delivery (181, 182). Additionally, studies suggest that combining Prussian blue nanoparticles with photothermal therapy and anti-CTLA-4 therapy may effectively treat neuroblastoma, improving survival and preventing recurrence (183). These findings highlight HT’s immunomodulatory potential, particularly in tumors that are otherwise resistant to ICI therapy.

### 8.4 Clinical evidence and ongoing trials

Several clinical trials are currently investigating the combination of HT and ICIs, with promising early results. Lyu et al. explored combining anti-PD-1 therapies (nivolumab and pembrolizumab) with thermal ablation in hepatocellular carcinoma (HCC) patients who had failed sorafenib treatment, leading to a significant improvement in the objective response rate (184). In biliary tract cancer, Xie’s application of tremelimumab combined with HT and ICIs achieved better progression-free survival (PFS) than second-line chemotherapy (185). Kleef’s research on stage IV triple-negative breast cancer with lung metastases, combining HT with immunotherapy, improved patient outcomes (44). In patients with advanced melanoma, the addition of localized HT to ICI therapy resulted in higher response rates and longer progression-free survival (PFS) compared to ICI therapy alone (186). Wei’s study on non-small cell lung cancer (NSCLC) patients using camrelizumab with microwave ablation also demonstrated promising outcomes (187). These findings suggest that the combination of HT and immunotherapy holds significant potential in clinical practice, though larger trials are necessary to refine treatment protocols and optimize patient selection.

### 8.5 Challenges and future directions

Despite these encouraging findings, several challenges remain in the clinical application of HT combined with ICIs. One of the main concerns is optimizing the timing and dosage of HT to maximize its immunomodulatory effects while minimizing toxicity. Another challenge is identifying biomarkers that can predict which patients are most likely to benefit from this combination therapy. Ongoing research is focused on refining HT techniques, including the use of nanotechnology to deliver targeted heat to tumors. Researchers are also exploring strategies for both whole-body and localized HT, as well as the potential of combining HT with other immunotherapeutic approaches, such as cancer vaccines and adoptive cell therapies. HT in combination with ICIs represents a promising approach to enhancing anti-tumor immune responses, particularly in tumors resistant to immunotherapy alone. As clinical evidence continues to accumulate, this combined approach may become an integral component of cancer treatment, offering new hope for patients with difficult-to-treat tumors.

## 9 Conclusions

Hyperthermia (HT) has emerged as a promising adjunct in cancer treatment, particularly in combination with immune checkpoint inhibitors (ICIs) and radiotherapy (RT). By modulating the tumor microenvironment, increasing immune cell infiltration, and promoting immunogenic cell death, HT can transform immunologically “cold” tumors into “hot” ones, enhancing their responsiveness to immunotherapy.

Future research should refine sequencing and dosing strategies for RT, HT, and immunotherapy to maximize synergy. Identifying predictive biomarkers such as HSP70, HSP90, and HMGB1 is crucial for improving patient selection and monitoring treatment efficacy (151, 188). Cytokines like IL-6 and TNF-α, as well as tumor-infiltrating lymphocytes (TILs), also serve as key indicators of immune activation (143). The TNFα-TNFR2 pathway, which regulates immunosuppressive and pro-angiogenic processes, warrants further investigation for its potential role in optimizing HT-ICI therapy (189).

A key research direction is comparing local HT and whole-body HT (WBH). Local HT improves tumor oxygenation and immune cell infiltration, while WBH enhances systemic immune responses and may amplify the abscopal effect. Future studies should evaluate their differential impacts on cytokine release, immune activation, and clinical outcomes to optimize treatment approaches.

All in all, integrating HT into multimodal cancer therapy requires further research into biomarker-driven patient selection and personalized treatment strategies. By enhancing immune checkpoint blockade efficacy and reshaping the tumor microenvironment, HT offers new therapeutic opportunities for patients with resistant tumors.
